# Species and condition specific adaptation of the transcriptional landscapes in *Candida albicans* and *Candida dubliniensis*

**DOI:** 10.1186/1471-2164-14-212

**Published:** 2013-04-02

**Authors:** Christian Grumaz, Stefan Lorenz, Philip Stevens, Elena Lindemann, Ulrike Schöck, Julia Retey, Steffen Rupp, Kai Sohn

**Affiliations:** 1 University of Stuttgart, IGVT, Nobelstr. 12 70569, Stuttgart, Germany; 2 Fraunhofer IGB, Nobelstr. 12, 70569, Stuttgart, Germany; 3 GATC Biotech, Jakob-Stadler-Platz 7 78467, Konstanz, Germany; 4 Genedata, Margarethenstr. 38 4053, Basel, Suisse

**Keywords:** Candida albicans, Candida dubliniensis, Transcriptional landscapes, RNA-Seq, Orthologs

## Abstract

**Background:**

Although *Candida albicans* and *Candida dubliniensis* are most closely related, both species behave significantly different with respect to morphogenesis and virulence. In order to gain further insight into the divergent routes for morphogenetic adaptation in both species, we investigated qualitative along with quantitative differences in the transcriptomes of both organisms by cDNA deep sequencing.

**Results:**

Following genome-associated assembly of sequence reads we were able to generate experimentally verified databases containing 6016 and 5972 genes for *C. albicans* and *C. dubliniensis*, respectively. About 95% of the transcriptionally active regions (TARs) contain open reading frames while the remaining TARs most likely represent non-coding RNAs. Comparison of our annotations with publically available gene models for *C. albicans* and *C. dubliniensis* confirmed approximately 95% of already predicted genes, but also revealed so far unknown novel TARs in both species. Qualitative cross-species analysis of these databases revealed in addition to 5802 orthologs also 399 and 49 species-specific protein coding genes for *C. albicans* and *C. dubliniensis*, respectively. Furthermore, quantitative transcriptional profiling using RNA-Seq revealed significant differences in the expression of orthologs across both species. We defined a core subset of 84 hyphal-specific genes required for both species, as well as a set of 42 genes that seem to be specifically induced during hyphal morphogenesis in *C. albicans*.

**Conclusions:**

Species-specific adaptation in *C. albicans* and *C. dubliniensis* is governed by individual genetic repertoires but also by altered regulation of conserved orthologs on the transcriptional level.

## Background

The most common fungal disease in man is candidiasis and is caused by several opportunistic *Candida* species. These *Candida* species are responsible for a whole set of diseases ranging from harmless superficial skin infections to deep-seated systematic candidiasis with relatively high mortality rates. Strikingly, the state of the host immune system determines the progression and severity of candidiases [1,2]. Therefore, systemic infections are predominantly found in patients with a compromised immune system. The most frequent and pathogenic species is *Candida albicans*[3,4]. One important characteristic associated with its virulence is the ability to switch between different morphologies [5]. This morphogenetic switch includes the transition from yeast form to true hyphae, the so called yeast-to-hyphae transition.

In 1995, Sullivan et al. described *Candida dubliniensis* as the phylogenetically closest relative to *C. albicans*. However, this species differs significantly from *C. albicans* in its virulence, as judged by the lower carriage rate and prevalence [3]. Clinical studies with patients from Great Britain as well as *in vivo* experiments with infected mice showed that *C. dubliniensis* seems to be far less successful in colonizing the human host causing systemic candidiases [6-8]. In contrast, *C. albicans* forms true hyphae to a greater extent under many conditions, often directly associated with higher virulence compared to *C. dubliniensis*[8-10].

In 2009, Jackson et al. [11] compared both species on the genomic level and could define 168 versus 29 species-specific genes for *C. albicans* and *C. dubliniensis*, respectively. However, more than 50% of the *C. albicans* specific genes were dubious and *in silico* predicted with no experimental confirmation so far. In their approach they also showed 5569 orthologous gene pairs and a high colinearity of 98.1% with respect to synteny. Yet, these investigations were mainly based on *in silico* predictions deduced from the respective genomes.

With the advent of powerful deep sequencing technologies [12,13], the transcriptional landscape of *C. albicans* was analyzed by RNA-Seq more comprehensively, showing that the transcriptome of this yeast is more complex than previously assumed [14,15]. Further investigations in *Saccharomyces cerevisiae* also revealed many noncanonical transcripts and alternative polyadenylation sites, which has not been described for yeasts before [16]. Thus RNA-Seq approaches provide promising tools for annotating and quantifiying whole transcriptomes experimentally [17-20].

In this context, we applied recent techniques in the field of RNA-Seq for annotating the transcriptional landscapes not only for *C. dubliniensis* but also for *C. albicans* to gain a solid and unbiased basis for the cross-species comparison regarding the genetic repertoires and their regulation. We generated two databases comprising of transcriptional units expressed under hyphal and yeast growth conditions using long read sequences from normalized and pooled cDNA fragments as well as short sequence reads from not normalized cDNA fragments, also used for quantification of the transcriptomes. In addition, we performed a cross-species comparison of their genetic repertoires to illustrate not only orthologous gene pairs and species-specific genes at the qualitative level but also the regulation of conserved genes at the quantitative level and thus, to define differentially expressed orthologs (DEOs) between both species. Accordingly, qualitative and quantitative differences identified in the transcriptional landscapes of *C. albicans* and *C. dubliniensis* might provide novel insights to explain the divergence in morphogenesis and hopefully offer a better understanding of the evolutionary adaptation of both fungi.

## Results

### Complementary deep sequencing technologies enable stringent gene annotations in *C. albicans* and *C. dubliniensis*

For an experimental annotation of the *C. albicans* and *C. dubliniensis* transcriptomes, we analyzed both fungi grown in two morphologies-blastospores and hyphae. Strikingly, the induction conditions for *C. dubliniensis* are quite harsh to form true hyphae while hyphal growth of *C. albicans* can be induced under a broad range of conditions, including YPD supplemented with 10% fetal calf serum (FCS) at 37°C (Additional file 1). Under this condition, *C. dubliniensis* remains in the blastospore form while *C. albicans* forms hyphae. Only in nutrient-poor environments like water supplemented with 10% FCS, *C. dubliniensis* grows as true hyphae. In this context, it is not yet clear why *C. albicans* can form hyphae while *C. dubliniensis* remains in the blastospore morphology under identical conditions, although both are phylogenetically so closely related. One possibility might be that the respective genetic repertoires are significantly different or that conserved genes are regulated in a different manner. To address these questions, we analyzed both transcriptomes on a qualitative as well as on a quantitative level.

**Figure 1:**
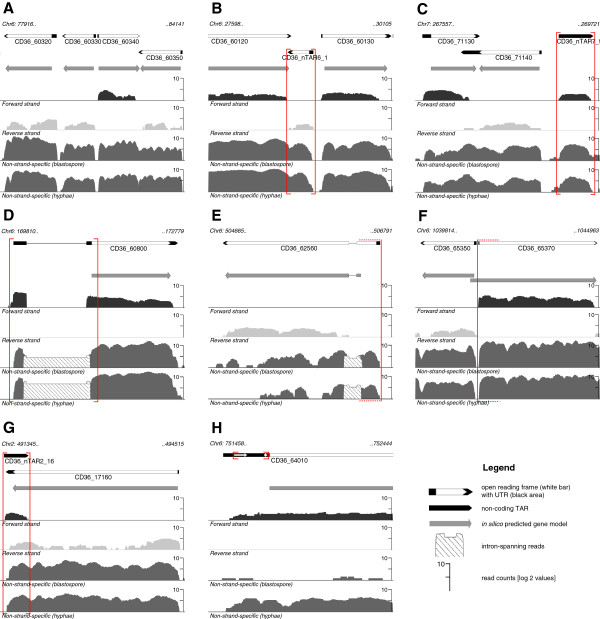
**Experimental annotation of transcriptionally active regions (TARs) in *C. dubliniensis.*** Genomic plots are visualized using the GeneScapes genome browser (downloadable at “http://code.google.com/p/genescapes”). The first row shows the experimental annotations revealed by this study (black arrows filled with white arrows) in comparison to the in silico predicted gene models (grey block arrows) in row two. RNA-Seq data are illustrated as wiggle plots. The third (forward strand, black) and fourth row (reverse strand, light grey) represent mapped reads from FLX sequencing while those from Illumina sequencing are shown in the fifth (blastospore condition) and sixth row (hyphae condition) in dark grey. Alterations made to the in silico predicted gene models are indicated by red boxes. (**A**) Experimental validation of four in silico predicted genes including UTR regions. (**B**) Annotation of a coding novel TAR (nTAR) and (**C**) a non-coding nTAR. (**D**) Detection of a novel splice site in CD36_60800. Alteration of the CDS region with an elongated ORF in CD36_62560 (**E**) and a shortenend ORF in CD36_65370 (**F**) due to correction of start sites. (**G**) Antisense transcription in the 3^′^-end of CD36_17160. (**H**) Two predicted uORFs in the 5^′^-UTR of CD36_64010.

For this purpose, we applied two deep sequencing technologies, the FLX454 and the Illumina technology, as complementary experimental approaches. For sequencing, total RNA was isolated from blastospores and hyphae and was subsequently utilized to generate two different types of cDNA libraries: one normalized library per species comprising strand-specific fragments from both growth forms for FLX sequencing as well as not normalized, condition-specific cDNA libraries, consisting of shorter fragments for Illumina sequencing (Additional file 1). Sequencing runs and subsequent mapping of reads to the respective reference genomes are shown in Additional file 2. In summary, we could uniquely align 147 million out of 161 million reads for annotation purposes (mapping efficiency >91%, Additional file 2). To revise the *in silico* annotations and validate them with experimental data, we combined the normalized and strand-specific reads with the complementary, highly abundant short reads and visualized them using the GeneScapes genome browser (Figure 1). Consequently, each gene was curated manually, resulting in a most stringent and high-resolution annotation of each species’ transcriptome (Additional file 3).

Following completion of the annotation in both species, we were able to analyze the expression of the respective transcriptomes. In summary, we found 6016 expressed genes for *C. albicans* and 5972 for *C. dubliniensis* of which 5541 and 5440 were coding and already annotated genes, respectively (Table 1, Figure 1A). The UTR length distribution across both species was highly similar (Additional file 4). Despite the observation that the median lengths of the 5^′^ UTRs (76 and 74 bp) are slightly shorter than in the 3^′^ UTRs (83 and 82 bp), there are still more longer 5^′^ UTRs with e. g. 180 genes containing UTRs longer than 500 bp compared to 3^′^ UTRs with only 60 genes in *C. albicans* (Additional file 5: Table S3A). GO term analyses of genes with longer 5^′^ UTRs than 500 bp in both species, showed significant enrichments for genes involved in many regulatory processes (Additional file 5: Table S3B-C). These data indicates a strong correlation between long UTRs and regulatory function in both fungal species as already described for *C. albicans* and *S. cerevisiae*[15,21].

**Table 1:** **Summary of transcript annotations in *C. albicans *and *C. dubliniensis***

	***C. albicans***				***C. dubliniensis***
	**This study**	***Sellam et al.***^***b***^	***Tuch et al.***^***b***^	***Bruno et al.***^***b***^	**This study**
Expressed genes	**6016**	*6574*	*(nd)*	*6608*	**5972**
Expressed annotated genes^a^ [coding]	**5541**	*4402*	*(nd)*	*6006*	**5440**
Median transcript length [bp]	**1400**	*(nd)*	*1237*	1305*	**1369**
5^′^ UTR median [bp]	**76**	*88*	*99*	80*	**74**
3^′^ UTR median [bp]	**83**	*84*	*136*	117*	**82**
nTARs	**475**	*2172*	*1437*	*602*	**532**
Coding nTARs	**26**	*11*	*561*	*13*	**70**
Novel introns	**79**	*(nd)*	*(nd)*	*41*	**108**
Different CDS	**262**	*(nd)*	*(nd)*	*(nd)*	**204**
Antisense transcripts	**210**	*729*	*759*	*(nd)*	**176**
*uORF* containing genes	**997**	*(nd)*	*(nd)*	*(nd)*	**1003**

nTAR = novel transcriptionally active region.

^a^ Reference genomes for *C. albicans* from CGD [2010-06-14] and for *C. dubliniensis* from NCBI [2010-04-09].

^b^ Data taken from orginal publications with their corresponding definitions, recalculated* or data could not be extracted *(nd)*.

Remarkably, we could measure transcription above background for more than 90% of all annotated genes by analyzing only two different conditions. Compared to data from Sellam et al. [22] where transcriptionally active regions (TARs) in *C. albicans* were detected by whole genome (tiling) microarrays out of four conditions which resulted in 4402 active coding genes, our sequencing approach seems to be more sensitive. In good agreement, two recent reports also showed highly sensitive detection of active transcripts by deep sequencing in *C. albicans*, e. g. detecting 6006 active coding genes across 13 conditions [14,15].

In addition to the experimental verification and refinement of already annotated genes, we found 475 and 532 novel TARs (nTARs) for *C. albicans* and *C. dubliniensis*, respectively (examples shown in Figure 1B,C). By applying stringent criteria for the determination of coding and non-coding nTARs (nc nTARs), we assigned 26 and 70 novel bona fide open reading frames (ORFs) for *C. albicans* and *C. dubliniensis*, respectively (Additional file 6). Thus, most of the novel TARs are non-coding. Bruno et al., Sellam et al. and Tuch et al. detected significant numbers of non-coding nTARs in *C. albicans* as well. We compared those with our nc nTAR datasets resulting in a core set of 69 nc nTARs annotated by all working groups strongly suggesting that these genes are true positives, 259 nc nTARs are consistent with at least one other group and 121 nc nTARs seem to be specifically found in our approach (Additional files 7 and 8). Some of the nc nTARs also belong to snoRNAs containing C/D-Boxes (Additional file 9), as some of them were also described recently by Mitrovich et al. [23].

Furthermore, not only for nTARs but also for already annotated genes we found 79 novel introns in *C. albicans* as well as 108 novel introns in *C. dubliniensis* (example shown in Figure 1D). Most of these novel introns are located in 5^′^ UTRs of annotated genes and in nc-nTARs. Among the 79 novel introns in *C. albicans*, 28 were also among the 41 novel introns described by Bruno et al., indicating reliability and accuracy of our approaches. The 13 novel introns found by Bruno et al. but not in our study have not been found as in 5 cases we did not see any detectable signals for splicing, in 3 cases the *in silico* predictions could be validated with our data representing no novel introns, in 4 cases we detected splicing signals but which are not consistent with our annotation criteria and for 1 intron we could not find the corresponding nTAR.

As the resolution of our annotation is at single base level and each gene was individually inspected, gene borders and splice sites could be set quite accurately. Strikingly, several *in silico* annotated ORFs and their corresponding CDS had to be elongated or shortened (Figure 1E,F). For 25 intron containing genes in *C. dubliniensis*, the splice sites had to be adapted resulting in CDS changes, while this kind of alteration was only necessary for one gene in *C. albicans* (Additional file 3). In summary, 262 and 204 CDS were elongated, shortened or altered in *C. albicans* and *C. dubliniensis*, respectively.

By using strand-specific reads, we were able to determine the orientation for all nTARs. Among these nTARs, some were overlapping with coding and annotated genes (example shown in Figure 1G). This kind of overlapping and antisense (AS) transcription was already described by Yassour et al. [24] via deep sequencing for *S. cerevisiae* (1106 antisense transcripts) as well as by Sellam et al. via tiling arrays for *C. albicans* (729 antisense pairs). Overall, we found 210 and 176 overlapping antisense pairs for *C. albicans* and *C. dubliniensis*, respectively. However, using our data sets, we could not analyze condition dependent expression of these transcripts nor could we assign certain GO terms. The majority of these AS pairs belong to a group of nc-nTARs that fully or partially overlap with protein coding genes (Additional file 10).

In addition, we could detect upstream ORFs (uORFs) for about 1,000 genes per species in their 5^′^ UTRs. An example for a conserved uORF is given in Figure 1H showing *CD36_64010*, the ortholog for YLR224W in *S. cerevisiae*, for which the larger uORF was already described [25]. These often very short ORFs (translated into 3–8 amino acids) in mature transcripts are thought to either enhance or repress the translation process of the downstream ORFs [26].

### Qualitative comparison of transcriptomes across both species reveals species-specific genes along with conserved genes

While the genomes of *C. albicans* and *C. dubliniensis* are largely conserved, there are distinct differences across both species like various inversions, insertion-deletions, and transposition events which might affect also pathogen-related genes as already described [11]. These data also revealed many orthologous gene pairs and some species-specific genes based on *in silico* predicted annotations in both species at that time. By using our approach, we were also interested in a cross-species comparison of experimentally verified TARs as well as in quantitative differences of gene expression of the corresponding orthologs in both species. In order to compare transcript abundances across both species, we initially had to define conserved and orthologous gene pairs, and genes which might be specific for one species without a clear ortholog in the other species. For this purpose, we applied the databases comprising our experimental annotations and *in silico* predicted annotations, to make sure not to miss any orthologs which might not be expressed under the given conditions.

**Figure 2:**
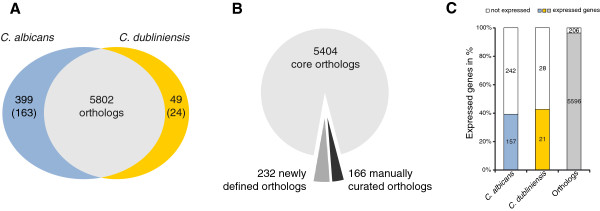
**Genetic repertoires of coding genes in *C. albicans *and *C. dubliniensis*.** (**A**) Coding genes of *C. albicans* and *C. dubliniensis* were compared at protein sequence level by reciprocal BLASTP. 5802 orthologous gene pairs versus 399 genes specific for *C. albicans* as well as 49 specific for *C. dubliniensis* were identified. Numbers in brackets indicate species-specific genes also reported by Jackson et al. (**B**) 5404 orthologs were consistent with the data from Jackson et al., 166 pairs had to be manually curated out of the raw blast results based on gene family information and on gene synteny. In addition to the current number of orthologous gene pairs between both species, we defined 232 novel pairs. (**C**) Fraction of expressed genes for species-specific and orthologous coding genes.

For all coding genes, we performed a reciprocal BLASTp search resulting in 5703 putative orthologs excluding (retro-) transposon associated genes and pseudogenes (Additional file 11: Table S8B), as well as 478 genes in *C. albicans* and 124 in *C. dubliniensis* which seem to have either no clear orthologs with reciprocal hits or even no hits at all (Additional file 11: Table S8C). As some of these genes belong to gene families or have gene duplicates, in some cases it is not sufficient to rely solely on homology of sequences but also on gene synteny. For this reason, we compared our orthologous pairs and species-specific genes with those of Jackson et al. for gene synteny and for gene families by individual inspection [11]. Accordingly, we found 5802 orthologs of which 5404 pairs were already determined by Jackson et al. and thus belong to the core set of orthologs (Figure 2B, Additional file 11: Table S8D). However, 166 pairs had to be manually curated out of the raw blast results based on gene family information and on gene synteny. In addition to the actual number of orthologous gene pairs between both species, we defined 232 novel pairs. The list of genes without clear orthologs was also revised, comprising 399 genes specific for *C. albicans*, 163 in agreement with Jackson et al. in addition to 236 novel specific genes, as well as 49 genes in *C. dubliniensis* of which 24 were also published by Jackson et al. and 25 novel species-specific genes (Figure 2A, Additional file 11: Table S8E). For this comparison every coding gene was taken into account (except pseudogenes and retrotransposon associated genes), even those genes for which we did not measure significant transcriptional levels (respective genes were maintained as *in silico* predicted genes in our annotation). Among the 5802 orthologs, 206 were not expressed in both species (3.6%), indicating that 96.4% (5596 gene pairs) were transcribed under the tested conditions. However, considering the proportion of expressed genes among the species-specific genes (157 out of 399 genes in *C. albicans* and 21 out of 49 in *C. dubliniensis*), the number decreases to roughly 40% (Figure 2C). The vast majority of the species-specific, non-expressed genes represent *in silico* predicted genes without any experimental or describing data so far. Thus, to check whether those genes are not expressed under the conditions tested or they just emerged from misannotations, we analysed the degree of conservation in eight closely related fungi. Indeed, it was lower for the non-expressed fraction than for the expressed fraction in *C. albicans* as well as in *C. dubliniensis* (Additional files 12 and 13). Additionally, the gene lengths of the expressed fractions seem to be visibly shorter than those of the non-expressed fractions-especially for *C. albicans*-specific genes (Additional file 14).

Taken together, the fraction of expressed, species-specific genes is significantly lower than the fraction of conserved genes which comprises the vast majority of all expressed genes under the conditions tested. Similarly, we performed reciprocal BLASTn searches for ncRNAs resulting in 131 non-coding, conserved orthologous pairs across both species (Additional file 11: Table S8F). 318 and 331 nc-nTARs seem to be specific for *C.alibicans* or for *C. dubliniensis*, respectively (Additional file 11: Table S8G).

### Cross-species comparison for differential expression of orthologs (DEO)

Using deep sequencing as an unbiased and open technology for gene expression profiling, it becomes possible to directly compare transcript abundances of e. g. conserved genes across two or more species. In this context, we were interested in the differential expression of the coding and non-coding 5933 orthologs (= DEO) in *C. albicans* and *C. dubliniensis* under one identical condition, including coding and non-coding genes. For this reason, we used transcript data from YPD supplemented with 10% FCS where *C. albicans* seems to follow different or additional adaptation pathways as *C. dubliniensis* which is reflected by their different morphologies. Therefore, it seemed likely that the expression of either species-specific or conserved genes is significantly different across both species. To test this hypothesis, we performed a cross-species gene expression analyses by plotting the ratio of normalized transcript abundances in both species against total abundance as MA-Plot for orthogonal expression.

**Figure 3:**
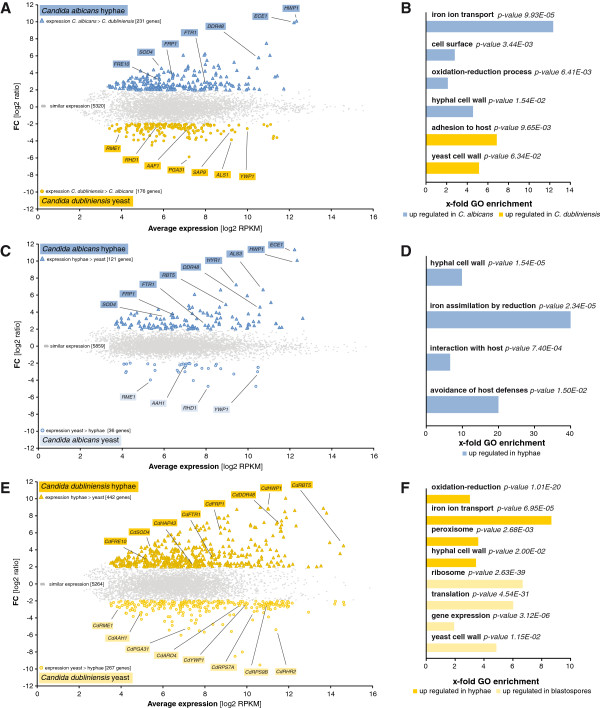
**Differential gene expression analysis in *C. albicans *and *C. dubliniensis.*** (**A**) Differential expression of orthologs (= DEO) between *C. albicans* and *C. dubliniensis* in YPD supplemented with 10% FCS. Blue triangles represent orthologous pairs with a significantly higher expression in *C. albicans* than in *C. dubliniensis* while those marked with yellow circles show higher expression in *C. dubliniensis*. (**C**) Yeast to hyphae (Y-t-H) switch in *C. albicans*. Blue triangles represent hyphally up regulated genes in YPD supplemented with 10% FCS while down regulated genes are marked with blue circles. (**E**) Yeast to hyphae (Y-t-H) switch in *C. dubliniensis*. Yellow triangles represent hyphally up regulated genes in water supplemented with 10% FCS while down regulated genes are marked with yellow circles. All marked genes are significantly differentially regulated with an adjusted p-value < 0,001 and a |FC| > 4. FC = fold change. (**B**, **D**, **F**) GO enrichment analysis of differentially expressed genes from the DEO analysis (**B**), the yeast to hyphae (Y-t-H) transition analysis in *C. albicans* (**D**) and in *C. dubliniensis* (**F**). Corresponding sets of up and down regulated genes were mapped with the “GO term finder” at CGD for two ontologies-biological process and cellular component. The x-fold enrichment is calculated as the ratio of percentages of the cluster frequency of the tested gene set and the cluster frequency of the genomic background. Representative GO terms were taken to illustrate trends of enrichment since most of the genes are assigned to more than one term.

Strikingly, 5320 out of 5727 experimentally verified and tested gene pairs are similarly expressed across both species showing comparable transcript abundances. On the contrary, 407 gene pairs (about 7% of all pairs) are differentially regulated, of which 231 pairs are significantly up regulated in *C. albicans* while 176 show higher transcript abundances in *C. dubliniensis* (Figure 3A, Additional file 15: Table S10B).

Assigning these genes with higher abundances in *C. albicans* to cellular processes or components, we found a significant enrichment of genes coding for cell surface proteins, especially from hyphal cell walls including the adhesin *HWP1* or the immunogenic stress-associated protein *DDR48* (Figure 3B, Additional file 16: Table S11A)*.* A process frequently co-regulated with hyphal growth in *C. albicans* is iron acquisition [27]. In this context, eight genes involved in iron ion transport were among the group of genes being up regulated in hyphal growing *C. albicans* including *FTR1*, *FRE10 *or *FRP1*. An example for the divergent regulation of hyphal-associated genes in both species is represented by *ECE1*. Though the function of *ECE1* is still unknown, its expression increases with hyphal cell elongation [28]. Our comparison shows that it is expressed at highest abundance level with a strongest fold change.

For blastospore expressed genes in *C. dubliniensis* grown in YPD supplemented with FCS at 37°C, a significant group could be assigned to biological adhesion processes with members like *YWP1, ALS1 or SAP9* (Additional file 16: Table S11B). Among the eight genes belonging to this GO, one transcription factor was identified, *AAF1*, which is involved in the regulation of adhesive cell surface proteins [29,30]. Another cell surface protein, *PGA31*, is assigned to the GO term for yeast-form cell wall composition and represents the ortholog with the highest difference in abundance among the group of genes having lower abundances in *C. albicans* than in *C. dubliniensis*.

To determine whether differential expression of these cell surface proteins and other differentially expressed orthologs either reflects species-specific adaptation in response to the environment or strictly correlates with morphology, we also analyzed the transcriptional profiles of *C. albicans* and *C. dubliniensis* during yeast to hyphae transition (Y-t-H), respectively.

For *C. albicans*, we compared the transcriptomes of hyphal growing cells in YPD supplemented with 10% FCS at 37°C with those of yeast growing cells in YPD at 30°C. Altogether, 157 genes were differentially regulated with 121 up regulated genes against 36 down regulated genes (Figure 3C, Additional file 15: Table S10C). Many of the orthologs we previously found by the DEO analysis with different expression rates between *C. albicans* and *C. dubliniensis* also came out to be differentially regulated during the intraspecies Y-t-H in *C. albicans*. For instance, a significant enrichment for orthologous genes coding for hyphal cell wall proteins was detected among the up regulated genes including *HWP1, DDR48* or *SOD4* (Figure 3D, Additional file 16: Table S11C). Other genes coding for iron assimilation proteins were likewise enriched (e. g. *FRP1* or *FTR1*). However, among those enriched groups *C. albicans*-specific genes has also been revealed like *HYR1* or *ALS3* coding for hyphal cell wall proteins. *ECE1* showed the highest fold change being up regulated in hyphal cells. Among the 36 down regulated genes we failed in assigning groups to certain GO terms. Rather, we detected individual genes including *YWP1* or *RME1*.

On the other hand, in *C. dubliniensis* hyphal morphogenesis is almost entirely linked to the adaptation to extremely harsh conditions like water supplemented with 10% FCS at 37°C. Thus, we compared the hyphal transcriptome with the yeast transcriptome in YPD supplemented with 10% FCS at 37°C also used for the cross-species comparison. We obtained 709 differentially regulated genes out of 5973 while 442 genes were up regulated under the hyphal condition and 267 genes were down regulated (Figure 3E, Additional file 15: Table S10D). For validation qRT-PCR was performed for 20 randomly selected genes from different classes of expression levels and fold changes resulting in a significant correlation of *r* = 0.98 (Additional files 17 and 18).

Among the most significant processes inhibited in water with serum is the translation machinery comprising 70 ribosome-associated proteins being down regulated in hyphae including the orthologs of the putative ribosomal proteins *RPS7A* (CD36_81410) and *RPS9B* (CD36_18490) (Figure 3F, Table S11F in Additional file 16). Accordingly, biosynthesis processes like the aromatic amino acid synthesis represented by *ARO4* (CD36_04870) or the glycerol biosynthesis represented by *RHR2* (CD36_80290) seem to be also strongly down regulated. This adaptation indicating deceleration of cell mass and cell proliferation is not surprising as the cells grow under an extreme nutrient poor condition.

**Figure 4:**
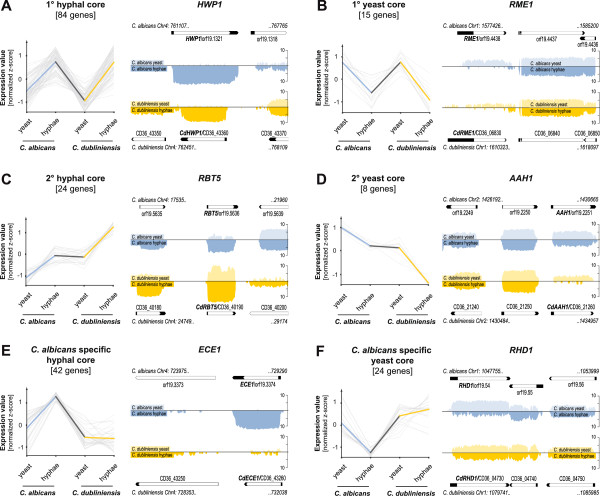
**Core sets of hyphal- and yeast-specific gene sets.** Expression profiles for the determined six gene sets each with one selected example representative for the corresponding profile visualized on the genome browser: (**A**) 1° hyphal core, (**B**) 1° yeast core, (**C**) 2° hyphal core, (**D**) 2° yeast core, (**E**) *C. albicans*-specific hyphal core and (**F**) *C.albicans*-specific yeast core. Gene sets were determined according to Additional file 15: Table S10E. Median expression values out of the three biological replicates are z-score normalized and plotted to the corresponding condition. Thicker lines represent the centroid profile. For visualization of gene expression for reference genes of each group, the GeneScapes genome browser is used. The orthologs *HWP1, RME1, RBT5, AAH1, ECE1* and *RHD1* are shown with their corresponding abundances. Scale as log2 values from read counts.

However, there are many up regulated genes during hyphal growth in *C. dubliniensis* with significant GO term enrichment in oxidation-reduction processes and in genes localized in peroxisomes (Table S11E in Additional file 16). Strikingly, among these genes, we detected eleven proteins involved in iron ion uptake including the orthologs of *C. albicans* for *FTR1* (CD36_13100), *FRE10* (CD36_43990) or *FRP1* (CD36_40170)*,* also identified by the DEO analysis between *C. albicans* and *C. dubliniensis*. Additionally, one of the orthologous key repressors for iron utilization, *HAP43* (CD36_10520), is up regulated as well. Cell surface genes were detected among both sets of differentially expressed genes including the hyphally induced (*HWP1* - CD36_43360, *DDR48* - CD36_23350) as well as the hyphally repressed genes (*YWP1* - CD36_22720, *PGA31* - CD36_43780). In order to identify core subsets of morphogenesis regulated genes which are conserved across both species and regulated during hyphal growth in a similar manner, we intersected hyphally up or down regulated genes from the Y-t-H analyses in *C. albicans* as well as in *C. dubliniensis* with those from the DEO analysis between *C. albicans* and *C. dubliniensis* (Additional file 15: Table S10E). Taken together, we determined six groups with characteristic expression profiles: 1° hyphal core, 2° hyphal core, *C. albicans*-specific hyphal core, 1° yeast core, 2° yeast core and *C. albicans*-specific yeast core (Figure 4).

Though the 1° and 2° cores share similarly up or down regulated genes in the respective species, the 2° cores show equal expression rates in the DEO analysis despite of divergent morphologies under identical condition. *RBT5* and *AAH1* are shown as representatives for the 2° hyphal core (24) and 2° yeast core genes (8) in Figure 4C and D, respectively. Amongst the 2° hyphal core, three genes are coding for cell surface proteins and two of these in turn play a role in heme-iron utilization-*PGA10* and *RBT5* (Table 2) [31]. It therefore seems that both 2° gene sets are not necessarily required for hyphal or yeast morphogenesis despite the fact of being up regulated during Y-t-H switches in both species.

**Table 2:** **Selected orthologs of *C. albicans *and *C. dubliniensis *differentially regulated**

**Cell surface/secreted**		
Hyphal core sets	No. of genes	Gene names*
1° in both species	16	*DDR48, ECM331, FRE10, FTR1, HSP70, HWP1, IHD1, PGA18,*
		*PGA23, PGA7, PHR1, PST2, RBR1, RBT4, SAP6, SOD4*
2° in both species	3	*RBT5, PGA10, PGA44*
*C. albicans*-specific	5	*DEF1, ECE1, PLB1, PHO113, PGA45*
Yeast core sets	No. of genes	Gene names*
1° in both species	3	*ALS1, PIR1, YWP1*
2° in both species	1	*SSA2*
*C. albicans*-specific	2	*SCW11, SAP7*
**Iron ion acquisition**		
Hyphal core sets	No. of genes	Gene names*
1° in both species	5	*CTR1, FRE10, FRP1, FTR1, orf19.7077*
2° in both species	3	*HMX1, RBT5, PGA10*
*C. albicans*-specific	0	-
Yeast core sets	No. of genes	Gene names*
1° in both species	0	-
2° in both species	0	-
*C. albicans*-specific	1	*ATM1*
**Transcriptional regulation**		
Hyphal core sets	No. of genes	Gene names*
1° in both species	3	*PST1, PST2, RFX2*
2° in both species	1	*BRG1*
*C. albicans*-specific	4	*UME6, SFL2, SET3, ZCF39*
Yeast core sets	No. of genes	Gene names*
1° in both species	3	*FCR1, NRG1, RME1*
2° in both species	0	-
*C. albicans*-specific	0	-

* Gene name aliases from CGD representing the corresponding orthogonal gene pairs from both species.

On the contrary, the 1° cores show clear profiles directly correlating with morphology, including the DEO analysis. 16 genes in the 1° hyphal core containing 84 genes are involved in cell surface architecture, like the orthologous pairs for *HWP1* (Figure 4C) and *DDR48,* five genes are involved in iron homeostasis and acquisition including *FTR1*, *FRE10* and *FRP1* and three genes are playing a role in regulation of DNA transcription, amongst others *RFX2*, a previously described hyphal-specific target of *NRG1*[32] (Table 2).

In addition, a smaller group of 15 genes was detected which seems to be hyphally repressed comprising the 1° yeast core subset of genes including three cell surface genes *ALS1*, *YWP1* and *PIR1* (Table 2) which were already described to be specific for yeast growing cells [33,34]. Strikingly, three transcription factors belong to this group as well—two known negative regulators (*FCR1* and *NRG1*) [32,35] and one yet uncharacterized gene (*RME1)* in *C. albicans* but known to be a meiotic regulator in *S. cerevisiae* (Figure 4B) [36].

However, there are also differences in the regulation of cell surface genes and of transcriptional regulators during hyphal growth between both species as well. Within the *C. albicans*-specific hyphal core set of genes, we could determine five cell surface genes specifically expressed in *C. albicans* under the hyphal-growing condition in YPD with serum, including *ECE1* (Figure 4E, Table 2)*,* the most abundant gene expressed in hyphae next to *HWP1*. In contrast, under hyphal-growing condition in *C. dubliniensis*, there was almost no transcription detected for the respective ortholog (Figure 4E). Beside these cell surface genes, four transcriptional regulators were found to be specifically up regulated in hyphal-growing *C. albicans*, e. g. *UME6* and *SFL2* which are known to be divergently regulated across both species [37,38]. In summary, we found 42 genes with an expression pattern comparable to *ECE1* or *UME6* (Figure 4E) representing differentially regulated orthologs with respect to morphogenetic adaptation. Vice versa, another 24 genes were classified as *C. albicans*-specific yeast core genes showing a profile like *RHD1* (Figure 4F), a putative mannosyl-transferase which is repressed during hyphal development in *C. albicans*[39] but obviously not in *C. dubliniensis*.

Taken together, these molecular insights provide evidence, that in addition to a core set of hyphally regulated genes, also species-specific sets of hyphally induced or repressed factors exist for *C. albicans* and *C. dubliniensis*, indicating that hyphae in both species are equipped in a different manner to adapt specifically to the respective environments.

## Discussion

Next-generation DNA sequencing technologies including RNA-seq provide unbiased approaches for the analyses of gene expression profiles. In contrast to DNA-microarrays where prior information about genomic annotations is essential, RNA-Seq conceptually is unbiased. Furthermore, the determination of quantitative data using RNA-seq is accomplished by counting individual transcripts rather than by deducing transcript abundances from signal intensities following hybridization to specific probes in microarrays. This concept with its open and unbiased architecture allows not only for experimental (*de novo)* annotations of whole transcriptomes but also provides the opportunity to analyze gene expression profiles across closely related species by directly comparing the normalized abundances determined as RPKM values of previously defined orthologs.

The combination of two complementary sequencing technologies (Illumina and FLX) for the experimental annotations in *C. albicans* and *C. dubliniensis* in our study resulted in a highly stringent definition of experimentally verified (>90% of the *in silico* predicted genes) as well as novel TARs. In this context, the rate of determining false positive TARs was minimized in terms of considering only TARs represented by reads above threshold and provided by both sequencing technologies. Bias intrinsic to one or the other technology thus was largely reduced. Approaches from other groups for the experimental annotation of transcriptional landscapes in *C. albicans* revealed far more novel TARs than we found, ranging from 1.437 nTARs using tiling arrays up to 2.172 nTARs using ABI Solid sequencing [14,22]. In contrast, Bruno et al. reported 602 nTARs generated using Illumina sequencing that more closely resembles the number we found with 475 nTARs. This might be due to the fact that we also used Illumina and by combination with FLX sequencing as well as applying stringent criteria for annotation thus resulted in the least number of nTARs. In fact, we also defined a core subset of 69 nc nTARs detected by four independent working groups and four independent technologies strongly arguing for the increasing importance of novel TARs, especially of non-coding nTARs. Nevertheless, we are sure that our study lacks a significant number of nTARs as our approach was optimized for higher specificity to reduce the number of false positives opposed to higher sensitivity that would result in a minimal number of false negatives.

Furthermore, it is obvious that the analysis of only a limited set of conditions cannot reveal the complete picture. However, based on the fact that using our approach we could detect 95% of already *in silico* annotated genes one might assume that we possibly missed about 5-10% of novel TARs. For *C. dubliniensis*, there have only been a few studies about global gene expression profiles since genomic sequence is available only previously [37,38,40]. In this context, our annotation might also lack for undetected genes in *C. dubliniensis* that are not expressed under the conditions tested.

In both species, we defined 96 novel protein coding genes of which 70 correspond to *C. dubliniensis*. Most of these genes contain smaller ORFs and were found to have orthologs in *C. albicans* which have been just recently added to the genome based on comparative genomic studies of eight *Candida* species by Butler and colleagues [41]. However, this study did not include the genome of *C. dubliniensis* explaining the higher number of novel coding genes in *C. dubliniensis* and the lower number of 26 novel coding genes in *C. albicans*.

An additional advantage of using experimental data generated by RNA-Seq is evident by the annotation of TARs at single nucleotide resolution. In both species many CDS had to be altered in terms of elongating and shortening them or containing amino acid exchanges due to incorrect predicted splice sites (taken together 466 genes). In some cases, elongations at the N-termini of proteins may contain additional functional domains which were missing recently reflecting the importance of experimental validation of *in silico* predictions.

However, the largest amount of novel genes belongs to the class of non-coding TARs comprising some snoRNAs and a significant number of antisense transcripts. Whether this observation provides a biological mechanism for post-transcriptional regulation like repression of translation or RNA-turnover remains to be analyzed. A recent study in *Schizosaccharomyces pombe* has shown that antisense RNAs are associated with several meiotically induced genes by repression of basal expression of the genes. And it has also been shown that the RNAi machinery is not necessary for this repression [42]. However, if such a mechanism is conserved in fungal species, it should represent a more general principle not restricted to the regulation of meiotic genes as many fungi including *C. albicans* and *C. dubliniensis* are lacking a meiotic cycle.

There is another transcript feature in post-transcriptional regulation which has been well known for *S. cerevisiae* for almost thirty years, the so called upstream ORFs in 5^′^ UTRs [43,44]. For each of the analyzed species, we found about 1,000 putative uORF containing genes with at least one uORF. This observation implies that the expression of many genes is far more complex than previously thought.

Qualitative comparisons between *C. albicans* and *C. dubliniensis* have already been performed in a previous study at the *in silico* prediction level revealing many coding orthologs (5569 pairs) as well as coding species-specific genes in both species-168 in *C. albicans* and 29 in *C. dubliniensis*, respectively [11]. While this study is exclusively based on the predicted gene annotation at that time, our experimental data is in good agreement to these results but further increases the number of respective genes assigned to the different categories. The majority of orthologs (5404 gene pairs) in both species is largely overlapping between our results and those from Jackson et al. and therefore might be considered as reliable. Nevertheless, with the addition of newly identified genes to the respective genetic repertoires and by inspections of gene syntenies on single gene level, we could further increase the number of orthologous gene pairs to 5802-including non-coding nTARs even up to 5933. In addition, our approach revealed about two times more species-specific coding genes in each species-399 and 49 in *C. albicans* and *C. dubliniensis*, respectively. However, only 40% of all species-specific genes were expressed above threshold levels, as only 63 out of 168 species-specific genes reported by Jackson and colleagues are expressed under the hyphae inducing condition we tested in *C. albicans*, for example. In contrast to the expression rate of 96% above threshold for those genes with clear orthologs in both species, this might implicate that although *C. albicans* and *C. dubliniensis* are equipped with unique sets of species-specific genes, they might not express them to that extent as revealed for orthologous genes. The reason for that might be, that they are simply not expressed under the tested conditions or that they represent *in silico* misannotations. At least, the lower degree of conservation of the non-expressed genes supports the latter. Remarkably, there is also a significantly high number of species-specific non-coding nTARs is for both species (with ~ 300 nc nTARs per species). However, not much is known about the function of non-coding TARs in *Candida* spp. and thus has to be investigated in ongoing studies.

While many potential novel targets have been revealed by the qualitative comparison of gene repertoires, we also focused on quantitative differences in the regulation of the orthologs comprising more than 90% all genes. In fact, the concept of RNA-Seq allows for the analyses of quantitative gene expression profiles across two different species. This study is the first to show the possibilities of differential expression of orthologs (DEO) by RNA-Seq and demonstrates a promising approach for unbiased comparisons across two species at very high resolution. A recent approach for comparative transcript profiling was described by O’Connor and colleagues based on *C. dubliniensis*-specific microarrays [37]. In contrast to O’Connor’s approach where hyphal-specific genes were determined by comparison of fold change ratios derived from microarray data of different labs, the main difference of our approach lies in the additional information revealed by the comparison of transcript abundances between different species under identical conditions rather than to exclusively utilize fold changes between different conditions and species. Our direct comparison of normalized abundances of all orthologous gene pairs revealed about 400 genes with significantly different abundance levels under one identical condition (YPD supplemented with serum). Thus, the adaptation of both species and the induction of e. g. hyphal growing *C. albicans* not only seemed to be dependent on the species-specific genetic repertoires but also on the divergent regulation of orthologous genes. Processes like iron uptake and utilization and hyphal cell wall composition are among the most significant differences between those species under the identical condition which directly correlates to their morphologies. Strikingly, iron uptake in turn is supposed to be tightly associated with hyphal induction and virulence as well as for persistence within the microbiome of the host [45]. Though we found many iron-responsive genes with higher transcription levels in *C. albicans*, we were not able to detect significant differences in the levels of the recently revealed, central regulators of the iron homeostasis circuit [27]. Indeed, there is no reason for the cells to induce their iron uptake machinery as the medium represents an iron repleted condition. Therefore, the central transcription factors (*HAP43* and *SEF1*) share similar expression rates in both species as expected, but still the data in *C. albicans* show higher rates of downstream targets of these regulators for iron uptake including *FTR1, FRP1* and *CFL1*. This observation might indicate a more complex transcription network for an adapted need of hyphal growing *C. albicans* cells which is independant from iron availability and obviously not activated in yeast growing *C. dubliniensis* cells under identical conditions. This means in particular that the diverged regulation of iron-related genes seems to be strictly linked to morphogenesis in *C. albicans*. The intraspecies Y-t-H switch analysis in *C. albicans* confirmed this hypothesis with both sets of genes being up regulated-genes for iron ion uptake and genes already described to be specific for hyphal development. Remarkably, there is only a limited number of hyphae inducing conditions for *C. dubliniensis* like water supplemented with serum, that in most cases lack nutrients including iron [37]. As revealed by our gene expression analysis of hyphal growing *C. dubliniensis* under this condition, genes involved in iron uptake also show high expression levels. In this case, the transcription factor *HAP43* and its targets (*FTH1, RBT5, FTR1 and FRP1)* are up regulated*.* Thus, iron metabolism seems to be strictly linked to hyphal morphogenesis in both species in a conserved manner.

Moreover, a whole set of 84 genes with similar expression profiles could be defined (1° hyphal core) containing 16 cell surface/secreted proteins (*HWP1* and *DDR48,* the two most abundant transcripts) and three transcriptional regulators (*PST1, PST2 and RFX2*), for example. In another set of 42 genes which are exclusively expressed in hyphal growing *C. albicans* (*C. albicans*-specific hyphal core), we found another five cell surface/secreted proteins and four transcription factors. In fact, this group represents a significant *C. albicans*-specific fingerprint of genes which neither are regulated in a conserved manner nor are they linked to hyphal morphogenesis in *C. dubliniensis*. Especially the cell surface/secreted protein *ECE1* is one of the most abundant genes expressed correlating with hyphal extension. In contrast, another recent study reported transcript profiling data from many induction cultures in *C. dubliniensis*, including water with serum showing that *ECE1* and also *UME6*, one of the four detected transcription factors within this group, are up regulated during hyphal morphogenesis [37]. However, this difference might be due to the fact that different strains with different filamentation properties were used as we worked with *C. dubliniensis* strain CD36 (CBS7987) while they worked with Wü284. From this it becomes clear that there are even significant strain-specific regulations within one species like shown for *ECE1* or *UME6* in these two strains and it might be reasonable to study different strains per species. Among the four transcription factors exclusively induced in hyphal growing *C. albicans*, there is also *SFL2* which has been described in accordance to our data not to be up regulated during RHE infection with *C. dubliniensis* strain CD36 (not filamentous in this infection model) but obviously essential for hyphal formation in *C. albicans*[38]. In addition, we found two promising novel transcriptional regulators which have not been described yet associated with divergently regulated genes across both species-*SET3*, coding for a histone deacetylase and suggested to regulate white-opaque switching and morphogenesis [46] and *ZCF39*, coding for a zinc-cluster protein possibly regulating adherence factors [47]. Both regulators might serve as starting points for further studies of *C. albicans* specific adaptation.

## Conclusions

Taken together, this study for the first time reports the comparative experimental annotation of transcriptional landscapes of the two most closely related fungi *C. albicans* and *C. dubliniensis*. Similarities as well as differences in the respective genetic repertoires and in the expression of orthologs and species-specific genes indicate that adaptation and virulence of both fungi are at least regulated at the transcriptional level. In this context, this study might contribute towards a better understanding of how regulatory networks in both species divergently adopted during evolution.

## Methods

### Strains and experimental conditions

For the generation of databases and gene expression profiles we used the closely related *Candida* species *Candida albicans* (SC5314) and *Candida dubliniensis* (CD36/CBS7987) under two different morphological conditions. Using YPD-medium (20 g/L peptone, 10 g/L yeast extract, 2% w/v glucose) at 30°C *C. albicans* is growing as blastospore, whereas hyphal growth is induced at 37°C using also YPD-medium but supplemented with 10% fetal calf serum. This condition was also used for blastospore induction in *C. dubliniensis*, whereas hyphal growth was induced by ddH_2_O supplemented with 10% fetal calf serum.

### RNA preparation

For the isolation of total RNA, blastospore and hyphae inducing media were inocculated with 3 × 10^6^ cells of the overnight culture of *C. albicans* and *C. dubliniensis*, respectively. After 4h of incubation under the corresponding conditions, *C. dubliniensis* and *C. albicans* cells were harvested by centrifugation and immediately frozen with liquid nitrogen. Disruption was carried out using a Mixer Mill MM 200 (RETSCH) with a shaking frequency of 30/s. The resulting powder was resuspended in lysis buffer RLT (QIAGEN, Hilden, Germany) supplemented with 0.01% v/v of ß-mercaptoethanol. The extraction of total RNA was performed according to QIAGEN’s Mechanical Disruption Protocol for the isolation of total RNA from yeast using the RNeasy Midi Kit. After precipitation of the RNA by addition of 0.1 volume of 3M NaAc pH 5.3 and 2.5 volume of 100% EtOH, the concentration and integrity of total RNA was analyzed using the Agilent 2100 Bioanalyzer using the RNA Nano kit. All experiments for FLXTitanium- and Illumina-sequencing including validation with qRT-PCR were performed using identical samples of total RNA.

### Preparation of cDNA libraries with subsequent high-throughput sequencing

For the normalized cDNA libraries for FLXTitanium-sequencing, equal amounts of approximately 25 μg of total RNA per condition were pooled together per species. To get rid of genomic contaminants another purification step was performed using QIAGEN’s RNeasy Mini Plus Kit. From the pooled total RNA poly(A)^+^-RNA was prepared according to standard protocols [48]. First-strand cDNA synthesis was carried out with a N6 randomized primer. Then 454 adapters A (5^′^-CCATCTCATCCCTGCGTGTCTCCGACTCAG-3^′^) and B (5^′^-CTGAGACTGCCAAGGCACACAGGGGATAGG-3^′^) were ligated to the 5^′^ and 3^′^ ends of the cDNAs, respectively, to obtain strand-specificity of the transcripts. Additionally, the *C. albicans* and the *C. dubliniensis* samples were barcoded at the 5^′^-end of the fragments with 5^′^-CGAGAC-3^′^ and 5^′^-CGTCGT-3^′^, respectively. The cDNAs were amplified with 16 cycles of PCR. Normalization was carried out by one cycle of denaturation and reassociation of the cDNA. Reassociated ds-cDNA was separated from the remaining ss-cDNA (normalized cDNA) by passing the mixture over a hydroxylapatite column. After hydroxylapatite chromatography, the ss-cDNA was amplified by 9 PCR cycles.

For Titanium sequencing the cDNA in the size range of 500-700 bp was eluted from preparative agarose gels. Aliquots of the size fractionated cDNAs were analyzed by capillary electrophoresis with the Shimadzu MultiNA microchip system. The two normalized and barcoded cDNA libraries were pooled at equal amounts and were sequenced on a full flow cell with the Titanium chemistry resulting in about 500.000 reads per species.

Illumina sequencing was performed on two different machines-GAIIx and HISeq2000. The cDNA libraries for GAIIx-sequencing were generated according Illumina’s mRNA-Seq Sample Prep Kit protocol with the samples *C. albicans* YPS bR1/2, *C. dubliniensis* YPS bR1/2 and *C. dubliniensis* H_2_O + FCS bR1/2 (see also Additional file 2). Each of the six samples was loaded on one lane. Thus, the sequencing run was performed on a fully loaded flow cell with single-end 74-75 bp reads and resulted in approximately 30 mio. reads per sample. These reads were predominantly used for annotation purposes and later on also for differential gene expression profiling. The cDNA libraries for HiSeq2000 were generated according Illumina’s TruSeq RNA Sample Prep Kit protocol with the samples *C. albicans* YPD bR1/2/3, *C. albicans* YPS bR3, *C. dubliniensis* YPS bR3 and *C. dubliniensis* H_2_O + FCS bR3 (see also Additional file 2). Samples were sequenced with single-end 50 bp reads. This dataset with a sequencing depth of about 10 mio. reads per sample was exclusively utilized for differential gene expression profiling.

### Alignment of FLX-Titanium reads

First, the reads were assigned to each species by decoding the barcodes. After separation of the reads, the barcodes at the 5^′^-end and the remaining sequences from the B adapter at the 3^′^-end were trimmed. The processed reads were then blasted against the corresponding databases with BLASTN (ncbi-blast-2.2.23). During our studies, we exclusively used the sequence and annotation files from Assembly21 (latest update 2010-06-15, http://www.candidagenome.org/download/sequence/C_albicans_SC5314/Assembly21) at CGD and from Assembly CD36 (latest update 2010-04-09, ftp://ftp.ncbi.nih.gov/genomes/Fungi/Candida_dubliniensis_CD36_uid38659) at NCBI. Only unique and non-spliced reads with at least 90% identities over the whole length of the reads has passed the quality filter. For intron spanning reads we performed BLAT alignments (BLAT Suite 0.34). Out of these alignments, it was possible to extract the position, the orientation and the exon junctions for each read.

### Mapping Illumina reads

For annotation purposes, the 74–75 bp reads from GAIIx sequencing were mapped with SOAP2 (version 2.20) using a seed length of 40 bp and allowing up to 5 mismatches throughout the whole length of the reads. As SOAP2 is not compatible with intron-spanning reads we performed another mapping with the remaining unmapped reads with TOPHAT (version 1.3.1) to capture them as well. Out of these alignments, it was possible to extract the position and the exon junctions for each read. The 50 bp reads generated with HiSeq2000 were exclusively mapped with TOPHAT (version 1.4.1) using default settings.

### Genome-associated assembly

Following reference genome based alignment of reads and manual inspection, we used the genome browser GeneScapes to annotate transcriptional active regions (Lorenz et al., manuscript in preparation, downloadable at “http://code.google.com/p/genescapes”). The aligned reads were plotted against the respective genomes in separate rows showing strand-specific FLX-reads (unique and non-unique), unspliced (unique and non-unique) and spliced GAIIx-reads. By combining the reads with the given *in silico* predicted gene models, we were able to define each single gene’s borders in single nucleotide resolution for about 6.500 genes per species. Afterwards, each of the annotated TARs (= transcriptionally active regions) had to pass two background filters in terms of abundancy of the represented reads per TAR. Thus, expressed *in silico* predicted genes has been experimentally verified and novel TARs annotated, if they were represented by more than 4 FLX-reads and their expression was higher as the determined background level in RPKM (= reads per kilobase of exon model per million mapped reads) in at least one replicate. In very few cases for non-coding nTARs, the criteria with a minimum of FLX reads was rejected and instead of that a reciprocal hit in the other species of an nTAR which met both previously described criteria was taken for a second criteria. The background was determined by quantifying the intergenic regions for each sample and taking the value of the 95^th^ percentile as background expression (Additional file 2). For some genes, restriction tags had to be added which are shown and described in Additional file 3.

### Defining novel open reading frames

To define ORFs for the identified novel TARs, we translated each possible reading frame in the given direction into a protein sequence. These sequences were aligned to closely related genomes via TBLASTN (default parameters). These were the genomes of *C. albicans, C. dubliniensis, C. glabrata, C. parapsilosis, C. tropicalis, D. hansenii, S. bayanus, S. castellii, S. cerevisiae, S. mikatae and S. paradoxus*. Only hits with an E-value better than 10^−6^ passed the first filter. The second filter allowed only for CDS (= coding sequence) with a length higher than 25% of the nTAR’s whole length. In the next step, CDS were sorted out with hits in single organisms. Finally, the hit with its CDS were taken with the highest bit score.

### Qualitative comparison between *C. albicans* and *C. dubliniensis*

For a qualitative cross-species comparison at protein sequence level, we initially performed reciprocal WU-BLASTP (BLOSUM62, query sequence length 1000, target database size 1000000). Reciprocal hits under an E-value of 10^−13^ were considered as true hits and thus orthologs, the remaining genes as species-specific as they had no significant hits or the hits they had did not map back reciprocally. Thus, many proteins from different families were assigned to the group of species-specific genes due to lacking reciprocal hits. By manually comparing these critical genes and also some of the reciprocal hits with the data form Jackson et al. [11] and inspecting them visually on the genome browser for synteny, we were able to define stringent orthologous and species-specific genes.

For non-coding genes, we performed reciprocal BLASTN and used the same significant E-value of less than 10^−13^ for determining orthologous or species-specific genes.

### BLAST comparisons with nc nTARs

BLASTN searches for determination of shared nc nTARs in *C. albicans* were performed with default settings (version 2.2.25+). Reference nc nTARs were taken from previous studies [14,15,22].

### BLAST comparisons with coding species-specific genes

BLASTP searches to evaluate conservation of species-specific, coding genes were performed with default settings (version 2.2.25+). Reference annotations from strains *Candida tropicalis, Candida parapsilosis, Candida orthopsilosis, Candida lusitaniae, Lodderomyces elongisporus, Debaryomyces hansenii, Candida guilliermondii* and *Candida glabrata* were downloaded from CGD (date 2013-01-31).

### Quantification of RNA-Seq data

Quantification was carried out according to Mortazavi et al. [20]. Thus, transcript abundance is calculated as RPKM (= reads per kilobase of exon model per million mapped reads). For normalization we performed standard quantile normalization using R with the limma package and added 1 rpkm to each expression value due to statistical reasons. Testing for differential expression was conducted with the DEGSeq package developed for RNA-Seq data [49]. Here, we considered genes with an adjusted p-value < 0.001 [50] and a fold change <−4 or >4 as significantly differentially regulated between two conditions.

### GO enrichment analysis

For GO term enrichment analyses we applied the web application “GO term finder” available on the “Candida Genome Database” (CGD, http://www.candidagenome.org/cgi-bin/GO/goTermFinder). When testing orthologous gene pair lists between *C. albicans* and *C. dubliniensis* or *C. dubliniensis* gene lists alone, the *C. albicans* orthologs defined in our study were chosen. The background for the test was appropriately adjusted by excluding those genes found to be specific for *C. albicans* without *C. dubliniensis* orthologs.

### Quantitative Real Time PCR (qRT-PCR)

Reverse transcription reactions were performed using Transcriptor High Fidelity cDNA Synthesis Kit (Roche, Mannheim, Germany). All reactions were carried out according to the manufacture’s protocol using 1 μg total RNA and 2.5 μM Anchored-oligo(dT)_18_ Primer. Quantitative Real Time PCR analysis was performed using the Universal ProbeLibrary Technology (Roche) and the LightCycler 480 Instrument. Amplification assays with a corresponding set of target-specific PCR oligonucleotides combined with a suitable Universal ProbeLibrary hydrolysis probe for 20 different *C. dubliniensis* genes were designed with the web-based ProbeFinder software, available at Assay Design Center (http://www.universalprobelibrary.com). Oligonucleotides were listed in Additional file 18.

Real Time PCR experiments were carried out in a 20 μl reaction volume containing 1:20 diluted cDNA template, 1 × Light Cycler 480 Probes Master (Roche) and 0.4 μM of each forward and reverse primer and 0.4 μM of the corresponding UPL probe. The reaction was done in duplicates (technical replicates) for each of the 20 genes. The standard curve preparation for the estimation of the PCR efficiency of each assay was performed under the same conditions, but using 5 dilution steps of the cDNA ranging between 10^−1^ and 10^−5^, and water instead of cDNA as a negative control. The PCR was carried out according to the following PCR protocol: 95°C for 5 min; 60 cycles of 95°C for 10 seconds, 60°C for 15 seconds, and 72°C for 1 second, afterwards the PCR reaction was cooled at 40°C for 30 seconds. The analysis of the expression levels was determined using 2^nd^ derivative max method provided by the Roche software version 1.5.0. For the *Candida dubliniensis* transcriptome, the gene expression values were normalized to the housekeeping gene *CdTUB1* (CD36_34530).

### Accession number

RNA-Seq data from FLX 454 and Illumina have been submitted to the NCBI Gene Expession Omnibus (GEO) under accession number GSE41749.

## Competing interests

The authors declare that they have no competing interests.

## Authors’ contributions

CG and KS conceived and designed the experiments. CG performed the experiments. US provided FLX 454 sequencing. CG, SL, PS, EL and JR analyzed the data. CG and KS wrote the paper. KS and SR reviewed and edited the paper. All authors read and approved the final manuscript.

## Supplementary Material

Additional file 1: Figure S1 Experimental design for RNA-Seq of *C. albicans* and *C. dubliniensis.* Bar represents 20 μm. Click here for file

Additional file 2: Table S Mapping statistics. Click here for file

Additional file 3: Table S2A-B List of annotated genes. Click here for file

Additional file 4: Figure S2 3^′^- and 5^′^-UTR distribution in *C. albicans* and *C. dubliniensis.* Only genes with annotated UTRs were taken into account. Click here for file

Additional file 5: Table S3A-C UTR analyses. Click here for file

Additional file 6: Table S4 List of novel coding genes. Click here for file

Additional file 7: Table S5 Comparison of novel nc nTARs with reference nc nTARs. Click here for file

Additional file 8: Figure S2 Venn diagram of nc nTARs found in our study tested against three reference datasets449 nc nTARs in *C. albicans* found in this study were compared with three independently generated sets of nc nTARs from Bruno et al. (590 nc nTARs), Sellam et al. (2161 nc nTARs) and Tuch et al. (866 nc nTARs). 121 out of 449 of our defined nc nTARs did not match in no reference set. Click here for file

Additional file 9: Table S6A-B RFAM and Snoscan results of novel non-coding genes. Click here for file

Additional file 10: Table S7A-C List of antisense gene pairs. Click here for file

Additional file 11: Table S8A-G Reciprocal blast results comprising orthologs and species-specific genes. Click here for file

Additional file 12: Table S9A-B Conservation of species-specific genes across eight further related species. Click here for file

Additional file 13: Figure S3A-D Conservation of species-specific genes across eight further related species. Click here for file

Additional file 14: Figure S4 Gene length distribution of expressed and non-expressed genes. Click here for file

Additional file 15: Table S10A-D Quantification and gene expression analyses. Click here for file

Additional file 16: Table S11A-F GO term enrichment analyses. Click here for file

Additional file 17: Figure S5 Validation of RNA-Seq data by qRT-PCR for 20 genes in *C. dubliniensis* during yeast to hyphae transition. Click here for file

Additional file 18: Table S12 List of primers used in qRT-PCR. Click here for file
