# Supplementary figures and images for: Species and condition specific adaptation of the transcriptional landscapes in Candida albicans and Candida dubliniensis
Source: BMC Genomics. 2013 Apr 2;14:212. doi: 10.1186/1471-2164-14-212 (PMC3626586; doi:10.1186/1471-2164-14-212)

## *Candida albicans*

## *Candida dubliniensis*

YPD at 30°C

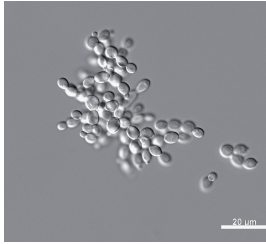

YPD + 10% FCS at 37°C

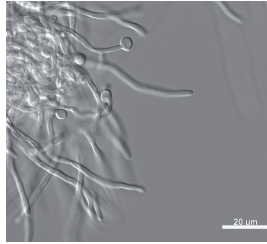

YPD + 10% FCS at 37°C

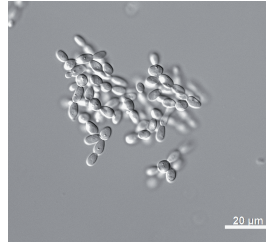

Water + 10% FCS at 37°C

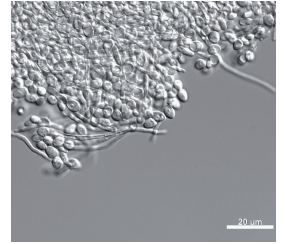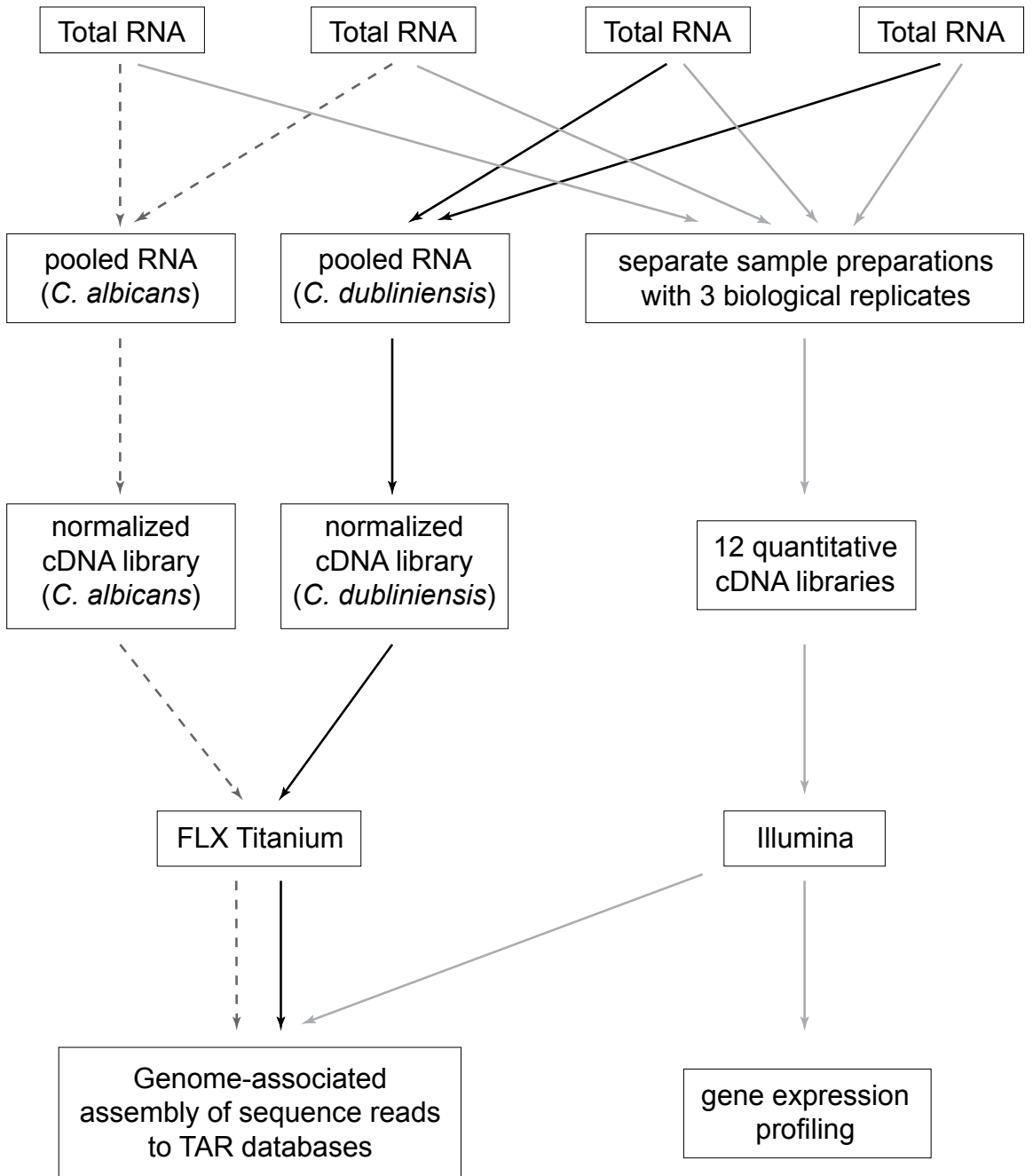

Supplement: Additional file 1: Figure S1 — Experimental design for RNA-Seq of C. albicans and C. dubliniensis. Bar represents 20 μm. [file 1471-2164-14-212-S1.pdf]

### 3'- & 5' UTRs in *C. albicans* & *C. dubliniensis*

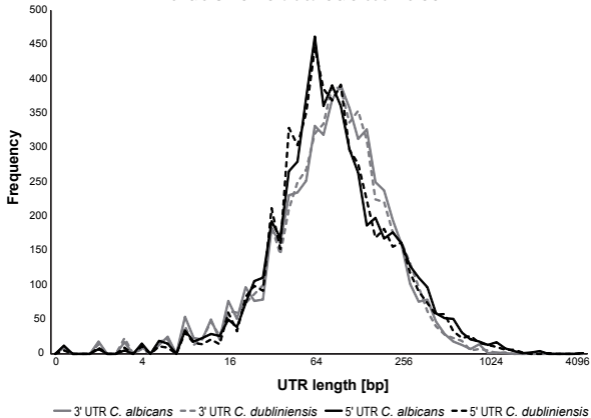

Supplement: Additional file 4: Figure S2 — 3′- and 5′-UTR distribution in C. albicans and C. dubliniensis. Only genes with annotated UTRs were taken into account. [file 1471-2164-14-212-S4.pdf]

**Tuch et al (866)**

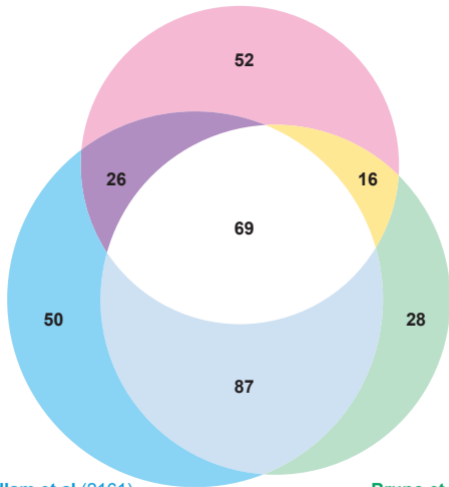

**Sellam et al (2161)**

**Bruno et al (590)**

Supplement: Additional file 8: Figure S2 — Venn diagram of nc nTARs found in our study tested against three reference datasets449 nc nTARs in C. albicans found in this study were compared with three independently generated sets of nc nTARs from Bruno et al. (590 nc nTARs), Sellam et al. (2161 nc nTARs) and Tuch et al. (866 nc nTARs). 121 out of 449 of our defined nc nTARs did not match in no reference set. [file 1471-2164-14-212-S8.pdf]

## *C. albicans*

■ expressed [157] ■ not expressed [242]

**A**

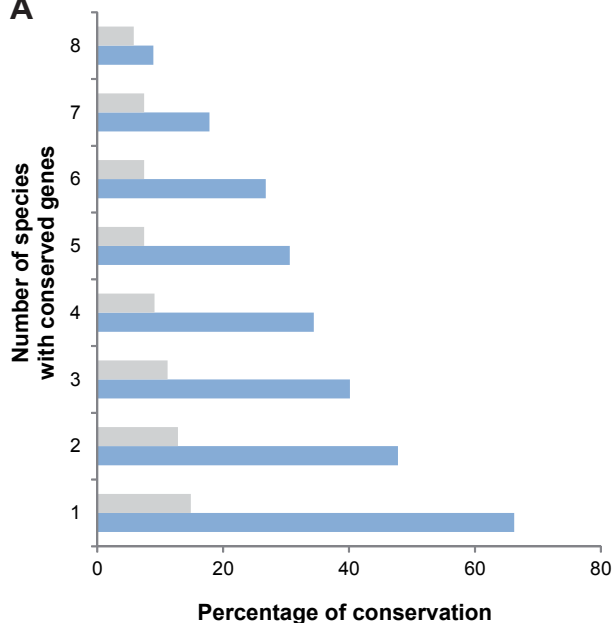

## *C. dubliniensis*

■ expressed [21] ■ not expressed [28]

**B**

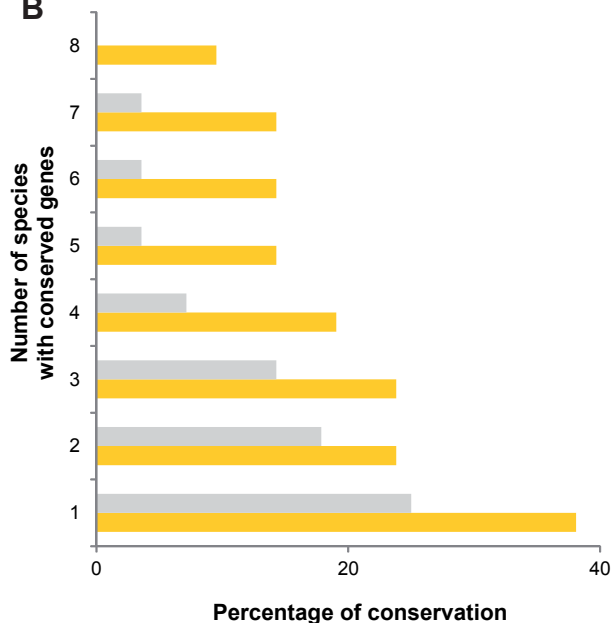

**C**

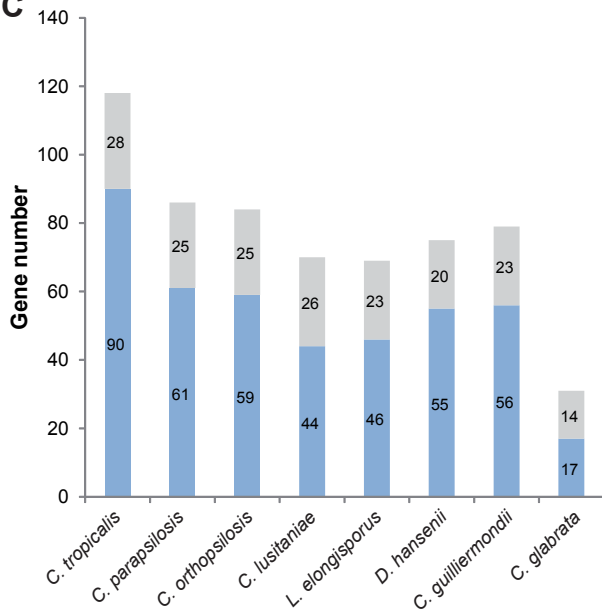

**D**

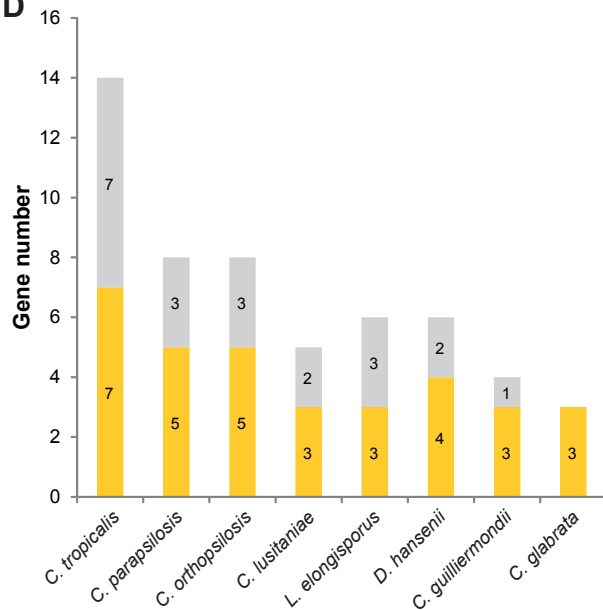

Supplement: Additional file 13: Figure S3A-D — Conservation of species-specific genes across eight further related species. [file 1471-2164-14-212-S13.pdf]

Gene length distribution [nt]

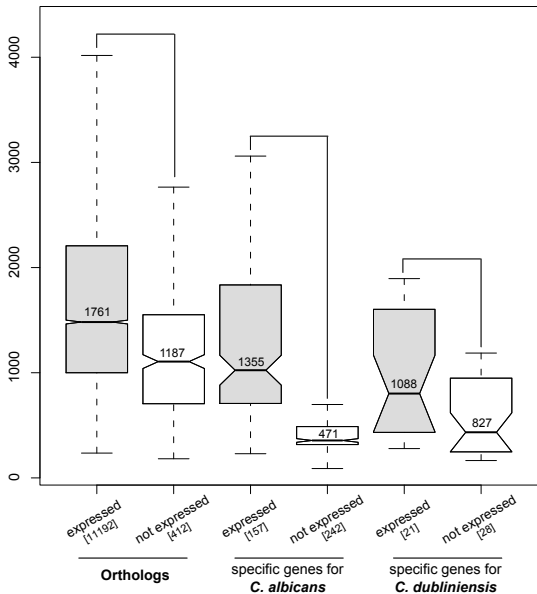

Supplement: Additional file 14: Figure S4 — Gene length distribution of expressed and non-expressed genes. [file 1471-2164-14-212-S14.pdf]

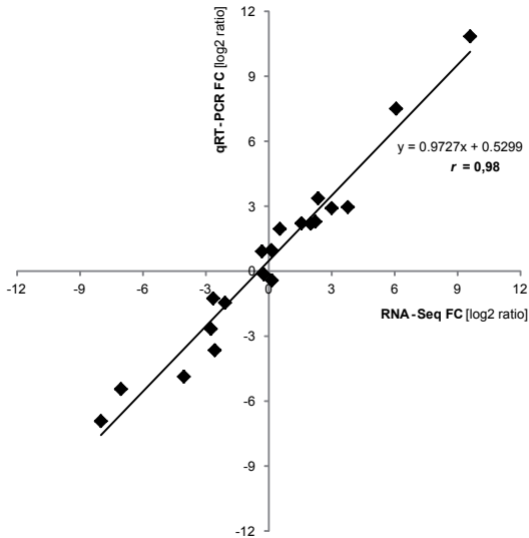

Supplement: Additional file 17: Figure S5 — Validation of RNA-Seq data by qRT-PCR for 20 genes in C. dubliniensis during yeast to hyphae transition. [file 1471-2164-14-212-S17.pdf]
